# Asthma-Like Features and Anti-Asthmatic Drug Prescription in Children with Non-CF Bronchiectasis

**DOI:** 10.3390/jcm9124009

**Published:** 2020-12-11

**Authors:** Konstantinos Douros, Olympia Sardeli, Spyridon Prountzos, Angeliki Galani, Dafni Moriki, Efthymia Alexopoulou, Kostas N. Priftis

**Affiliations:** 1 Allergology and Pulmonology Unit, 3rd Paediatric Department, National and Kapodistrian University of Athens, 12462 Athens, Greece; costasdouros@gmail.com (K.D.); ol.sardeli@googlemail.com (O.S.); angeliki.galani@gmail.com (A.G.); dafnimoriki@yahoo.gr (D.M.); 2 2nd Radiology Department, National and Kapodistrian University of Athens, Attikon University Hospital, 12462 Athens, Greece; spyttt@gmail.com (S.P.); ealex64@hotmail.com (E.A.)

**Keywords:** inhaled corticosteroids, non-CF bronchiectasis, asthma, children

## Abstract

Bronchiectasis and asthma may share some characteristics and some patients may have both conditions. The present study aimed to examine the rationale of prophylactic inhaled corticosteroids (ICS) prescription in children with bronchiectasis. Data of children with radiologically established bronchiectasis were retrospectively reviewed. Episodes of dyspnea and wheezing, spirometric indices, total serum IgE, blood eosinophil counts, sensitization to aeroallergens, and air-trapping on expiratory CT scans, were recorded. The study included 65 children 1.5–16 years old, with non-CF bronchiectasis. Episodes of dyspnea or wheezing were reported by 22 (33.8%) and 23 (35.4%), respectively. Skin prick tests to aeroallergens (SPTs) were positive in 15 (23.0%) patients. Mosaic pattern on CT scans was observed in 37 (56.9%) patients. Dyspnea, presence of mosaic pattern, positive reversibility test, and positive SPTs were significantly correlated with the prescription of ICS. The prescription of ICS in children with bronchiectasis is more likely when there are certain asthma-like characteristics. The difficulty to set the diagnosis of real asthma in cases of bronchiectasis may justify the decision of clinicians to start an empirical trial with ICS in certain cases.

## 1. Introduction

Bronchiectasis is a complex and progressive respiratory disorder, characterized by chronic infection, inflammation, and abnormal dilatation of the bronchi. The loss of bronchial wall integrity, the mucus impaction and mucosal oedema may reduce the lumen opening and restrict the airflow especially during expiration when the bronchial walls appose. Bronchiectasis is a syndrome and not a disease per se, and several causative and associated disorders have been described [1].

Children with bronchiectasis, apart from cough, may also develop wheeze and asthma symptoms. with reported rates ranging from 11 to 46% [2,3], although it is not always clear if this is a consequence of coexistent asthma, or is a direct result of bronchiectasis. The difficulty in clarifying this point is not only due to the scarcity of adequate data but also to the lack of a simple clinical tool to identify asthma in children and, the vagueness of the main clinical characteristics of asthma. Indeed, dyspnea is a subjective feeling of the patient [4] and wheezing can be easily confused with other respiratory sounds especially when its presence is reported by patients or parents [5]. However, there is no doubt that asthma and bronchiectasis do coexist in some patients, and despite the absence of clear information on a mechanism linking the two conditions, the apparent implication of their overlap is a more severe disease with more frequent exacerbations [6].

The treatment of bronchiectasis is based mainly on antibiotics and chest physiotherapy. In cases of bronchiectasis and asthma coexistence, patients must receive treatment for both conditions [7,8]. Nevertheless, in daily practice, inhaled corticosteroids, with or without long-acting beta-agonists (LABA), are frequently prescribed in patients with bronchiectasis even when there is no clear evidence of coexisting asthma. Although the reason that drives physicians to adopt this non-evidence based practice is not clear, it might be related to the limited treatment options, the variable and often non-satisfactory response to therapy, and the frequent exacerbations of bronchiectasis. The present study aimed to describe the extent of the empirical use of ICS in children with bronchiectasis and the rationale behind the implementation of this practice.

## 2. Methods

The present study was conducted in the Pediatric Pulmonology Unit of the Attikon University Hospital in Athens, which is one of the main tertiary referral centres for pediatric pulmonary disorders, in Greece. Three consultants and four fellows had been serving in the Unit during the study period. Data of all the children attended in the Unit from 2013 to 2018, up to 16 years old, with chronic wet cough and radiologically established bronchiectasis on chest high-resolution computed tomography (HRCT) scan were retrospectively reviewed. All HRCT scans had been performed in the radiology department of our hospital and were evaluated by the same pediatric radiologist who was aware of the patients’ clinical history. The criteria for the diagnosis of bronchiectasis on HRCT were dilatation of bronchi, as determined by broncho-arterial ratio > 0.80 [8,9]; parallel bronchial walls in a longitudinal section (tram sign); visualization of bronchi within 1 cm of pleura. We used the modified Bhalla score to quantify the severity of bronchiectasis [10]. The presence of areas of decreased attenuation on expiration (“mosaic attenuation”) suggestive of airways obstruction was recorded.

Investigations in all patients included a complete blood count, sweat test and/or cystic fibrosis (CF) gene mutation analysis, measurement of serum immunoglobulins, and skin prick tests (SPT) to the most common aeroallergens (olive, grass, Parietaria, Chenopodium, cypress, house dust mite, fungi, cat and dog dander). Nasal nitric oxide test and high-speed video microscopy analysis were performed only in patients who fit the clinical phenotype of primary ciliary dyskinesia. Spirometry was performed at the initial and the follow-up visits to all patients ≥5 years old who were able to cooperate. T_H_2-high status was defined as the combination of total serum IgE levels of ≥100 IU/mL and absolute blood eosinophil count of ≥140/mL [11].

All patients who reported episodes of dyspnea (shortness of breath) or wheezing, and/or their spirometric indices or the shape of the spirometry loop were indicative of airway obstruction, and/or had positive SPT, underwent a bronchodilator response (BDR) test with salbutamol, during their first visit. The BDR test was performed with four actuations of salbutamol metered dose inhaler into a spacer device and interpreted as positive if the change in the forced expiratory volume in one second (FEV1) after bronchodilator administration was ≥ 12%. Spirometric indices were reported as percent predicted (pp) values using the NHANES III reference equations. All patients received antibiotics for 3–6 weeks, started daily chest physiotherapy, and some of them were also prescribed ICS with or without LABA. All were reevaluated in 2–3 months.

Patients with CF were excluded from the study.

The study protocol was approved by the Attikon University Hospital Ethics Committee.

### Statistical Analysis

Variables are presented as mean with standard deviation (sd) or as median with interquartile range (IQR). Univariate analysis was performed with paired t-test, chi-square test, and Spearman’s rank correlation test with Bonferroni correction for multiple comparisons. Correlations were expressed as Spearman’s ρ with its corresponding 95% confidence interval (CI). The variables which in univariate analysis were significantly correlated with the decision to start ICS were included as explanatory variables in a multivariate logistic regression model. Results of multivariate analysis are presented as odds ratios (OR) with 95% confidence interval (CI). We further performed three linear regression models where ICS use was the explanatory variable and the differences in FEV1, FEV1/FVC, and the number of positive cultures, between the follow-up and the initial visit, were used as the response variables.

## 3. Results

Sixty-five children 1.5–16 years old, were diagnosed with non-CF bronchiectasis; 46 (70.7%) of them had been referred as cases of difficult asthma. Thirty-nine (60%) children had been referred by general pediatric practitioners, and 26 (40%) by pediatric departments and clinics. Sixteen (24.6%) patients had underlying disorders directly or possibly related with bronchiectasis: five had tracheoesophageal atresia, five had PCD, one had Crohn disease that was treated with infliximab, one had Job syndrome, one had congenital pulmonary airway malformation (CPAM), one had anhidrotic ectodermal dysplasia with immunodeficiency, and one had IgA deficiency. The remaining 49 (75.4%) patients had no identifiable cause. Apart from the child with the IgA deficiency, all children had normal for age serum concentrations of IgA, IgG, and IgM. Twenty-eight (43.0%) patients commenced daily treatment with ICS with or without LABA. Patients’ clinical characteristics and their correlation with the prescription of ICS are shown in Table 1. Spirometry results and bacteria isolated from sputum cultures at the first and second visit are shown in Table 2. Dyspnea, presence of mosaic pattern, positive SPT, and positive BDR test were significantly correlated with the prescription of ICS (Table 1).

The multivariate logistic regression model included as explanatory variables the presence dyspnea, positive SPT, and of mosaic pattern, and corroborated the correlations found in univariate analysis between these variables and the decision to prescribe ICS (OR:5.35; CI:1.11, 25.80; *p* = 0.036, and OR:10.89; CI:2.21, 53.23; *p* = 0.003, and OR:12.55; CI:1.21, 121.61; *p* = 0.034, respectively). BDR test was not included in the model due to its small sample size and the lack of variation that did not allow a reliable estimation of model parameters. The three regression models showed that ICS use was not correlated with the differences in FEV1, FEV1/FVC, and the number of positive cultures, between the follow-up and the initial visit (*p* = 0.29, *p* = 0.69, and *p* = 0.20, respectively).

## 4. Discussion

Many of the bronchiectatic patients in our study had clinical, and/or spirometry, and/or radiological findings indicative of airway obstruction which could be attributed either to coexisting asthma or to bronchiectasis per se. The lack of a simple test for the identification of asthma in children and the absence of strict criteria for ICS use in bronchiectasis, allow physicians to decide on the prescription of these drugs in a seemingly arbitrary way. Very often physicians base their diagnosis of asthma on a therapeutic trial of ICS and a careful assessment of the children’s response. Unfortunately, this approach is not always helpful in patients with bronchiectasis due to the confounding effect of co-administered treatments. Some authors have suggested that ICS should be reserved for children with evidence of type 2–mediated allergic airway inflammation, whose existence can be roughly determined or ruled out by the measurements of eosinophils and IgE with or without concurrent measurement of fractional exhaled nitric oxide (FeNO) [8,12]. However, asthma in children is a heterogeneous disorder and atopic inflammation is neither necessary nor sufficient for its development. So, the aforementioned markers cannot be considered as definitive diagnostic tools for asthma [13]. In the present study, the main findings that influenced the decision of ICS prescription were the reported episodes of dyspnea, the presence of mosaic pattern on HRCT, the positive SPTs to aeroallergens, and the positive BDR test.

Dyspnea and wheezing were the only clinical signs/symptoms that were included in our analysis as they represent the two most common characteristics reported by parents, or experienced by children, with asthma [14]; only dyspnea was shown to affect the decision for ICS prescription. A probable explanation is that dyspnea is a rather unusual symptom in bronchiectasis, with a reported incidence ranging from 8.8–25.0% [3,15], while it is very common in asthma. On the other hand, wheeze implies flow limitation and it is not an exclusive characteristic of asthma. In bronchiectasis, it can be produced from mucus hypersecretion leading to bronchostenosis and the collapse of bronchial walls during expiration. Furthermore, when patients/parents report wheeze it is far from clear what they actually describe since many use this term for any noisy breathing [5,16].

Atopy, which is a considerable risk factor for asthma development [17,18], was investigated through SPT’s to aeroallergens, as well as total serum IgE and absolute blood eosinophil count measurements. SPTs, which are strongly associated with respiratory allergy and asthma [19], were associated with ICS prescription. On the contrary, no significant association was found between ICS prescription and the serum IgE or eosinophil count, or their combination in a T_H_2-high status variable. This may have resulted from serum IgE and eosinophil count being less consistent markers of asthma and allergy due to the absence of specific cut off values and the considerable overlap between normal and allergic patients [18,20].

Spirometry results were not a significant determinant of ICS prescription in our population. Indeed, spirometric values are usually normal in asthmatic children who are not in exacerbation and so they are not a reliable marker of asthma [21]. On the contrary, a positive BDR test has 50% positive predictive value in identifying response to ICS in asthmatic children with normal spirometry [22], and in accordance with this, our study showed the test to be a significant predictor of ICS prescription.

The mosaic pattern in expiration HRCT scans reflects air trapping and it is a well-known radiological feature of bronchiectasis [23]. However, air trapping was correlated with ICS prescription as it also represents a characteristic of asthma, with studies in adults and children having shown that it has a strong relationship with disease severity and peripheral airway obstruction [24,25].

The present study has certain limitations. The data depict the prescription preferences of physicians from a single centre and so the results are neither free from bias nor can be generalized. Cough, one of the main features of asthma, was not assessed because it is also the cardinal symptom of bronchiectasis and as such it could not offer any discriminative information for asthma diagnosis. HRCT scans were evaluated by a single radiologist and were not blinded. All children received antibiotics and started chest physiotherapy at diagnosis. Because of this, we were unable to determine the net result, if any, of ICS in children with asthma-like features. We tried to examine whether the administration of ICS could result in any measurable clinical changes that would allow us to identify a group of children who could truly benefit from ICS. However, since the study was not designed to investigate this question, included only a few outcome variables, and the sample size was relatively small, the results were inconclusive.

In conclusion, it was shown that the prescription of ICS in children with bronchiectasis is more likely when there are certain asthma-like features. The results should be conceptualized as a justification of the clinicians’ decisions in cases where the coexistence of bronchiectasis and asthma seems probable and not as evidence for the need for ICS in certain cases. Indirectly, the study stresses the importance of research data able to illuminate the issue of asthma and bronchiectasis coexistence and define the clinical characteristics of children with bronchiectasis who could benefit from the use of ICS.

## Figures and Tables

**Table 1:** Correlations of the main clinical, radiological, and laboratory characteristics of the 65 bronchiectasis patients with inhaled corticosteroids.

			Correlation with ICS Use	
	Without ICS Use (*n* = 28)	With ICS Use (*n* = 37)	Spearman ρ (CI)	*p*
Male, *n* (%)	16 (53.3)	14 (46.6)	0.08 (−0.22, 0.33)	0.90
Age at referral, years, *n* (%)	7.5 (2.7)	7.7 (2.8)	0.04 (−0.19, 0.29)	0.86
SPT, *n* (%)	28 (100)	37 (100)		
‒ Positive SPT, *n* (%)	1 (2.7)	15 (53.6)	0.55 (0.36, 0.70)	<0.001
Serum IgE, IU/mL, mean (sd)	49.8 (47.7)	72.1 (69.4)	0.25 (−0.03, 0.45)	0.16
Blood eosinophil count, mean (sd)	260 (225)	361 (322)	0.26 (0.01, 0.40)	0.20
T2-high status, *n* (%)	3 (8.1)	3 (10.7)	0.04 (−0.09–0.39)	0.72
Spirometry, *n* (%)	22 (48.9)	23 (51.1)		
‒ ppFEV1, mean (sd)	94.2 (13.5)	88.5 (12.1)	−0.41 (−0.63, −0.14)	0.13
‒ ppFVC, mean (sd)	92.9 (20.2)	88.1 (23.8)	−0.25 (−0.09, 0.78)	0.49
‒ FEV1/FVC, mean (sd)	87.1 (7.9)	83.9 (8.9)	−0.37 (−0.62, −0.13)	0.25
Bronchodilator response test, *n* (%)	10 (34.5)	19 (65.5)		
‒ Positive bronchodilator response test, *n* (%)	0 (0)	11 (57.9)	0.57 (0.21, 0.92)	0.001
Reported symptoms				
‒ Episodes of dyspnea, *n* (%)	4 (10.8)	18 (64.3)	0.55 (0.36, 0.70)	<0.001
‒ Wheezing, *n* (%)	8 (21.6)	15 (53.6)	0.33 (0.09, 0.53)	0.31
Modified Bhalla score, mean (sd)	4.1 (2.4)	3.6 (2.3)	0.21 (0.08, 0.42)	0.25
Mosaic pattern on HRCT, *n* (%)	12 (32.4)	24 (85.7)	0.53 (0.33, 0.68)	<0.001
Exacerbations during the first six months of treatment, median (IQR)	2 (1,2)	2 (1,3)	0.13 (0.02, 0.28)	0.27

ICS: Inhaled corticosteroids; HRCT: High-resolution computed tomography; SPT: Skin prick tests; pp: percent predicted.

**Table 2:** Spirometry results and bacteria isolated from sputum cultures at the first and second visit of patients.

	Initial Visit (*n* = 65)	Follow-Up Visit (*n* = 65)	*p*
Spirometry, *n* (%)	45 (69.2)	45 (69.2)	1
‒ ppFEV1, mean (sd)	91.8 (20.4)	93.1 (19.1)	0.65
‒ ppFVC, mean (sd)	90.1 (23.3)	93.3 (24.6)	0.44
‒ FEV1/FVC, mean (sd)	84.4 (10.4)	85.6 (11.5)	0.50
Positive sputum cultures, *n* (%)	38 (58.4)	3 (13.8)	<0.001
‒ Gram-negative bacteria, *n*	12	2	
‒ Staphylococcus aureus, *n*	7	1	
‒ Pseudomonas aeruginosa, *n*	7	0	
‒ Haemophilus influenzae, *n*	5	0	
‒ Streptococcus pneumoniae, *n*	4	0	
‒ Others, *n*	3	0	
